# Incidence and Characteristics of Retinopathy of Prematurity Patients With Late Gestational Age and Large Birth Weight in South China

**DOI:** 10.3389/fmed.2022.712759

**Published:** 2022-03-03

**Authors:** Xiang Gao, Yunru Liao, Duoru Lin, Lisha Wang, Deying Yu, Zijing Li, Yichi Zhang, Yuqing Lan

**Affiliations:** ^1^Department of Ophthalmology, Sun Yat-sen Memorial Hospital, Sun Yat-sen University, Guangzhou, China; ^2^State Key Laboratory of Ophthalmology, Zhongshan Ophthalmic Center, Sun Yat-sen University, Guangzhou, China

**Keywords:** retinopathy of prematurity, late gestational age, large birth weight, vaginal delivery, characteristics

## Abstract

**Purpose:**

To investigate the incidence and characteristics of retinopathy of prematurity (ROP) premature infants with late gestational age (GA) and large birth weight (BW) and show a 7-year trend of ROP incidence in South China.

**Methods:**

This retrospective, cross-sectional study included premature infants who received ROP screening in a 7-year period (from 2010 to 2016) at the Sun Yat-sen Memorial Hospital (SYSMH), Guangzhou, South China. Infants were screened if they had GA <37 weeks or BW <2,500 g. All screened infants were divided into two groups: Group 1 (with both GA ≥ 35 weeks and BW ≥ 1,750 g) and Group 2 (others). The characteristics of ROP infants in Group 1 were analyzed and compared with those in Group 2.

**Results:**

A total of 911 premature infants were screened, with 282 infants in Group 1 and 629 in Group 2. Both the incidences of any ROP (6.7 vs. 8.3%, *p* = 0.50) and Type 1 ROP (1.4 vs. 1.7%, *p* = 0.72) in Group 1 were comparable with those in Group 2. Lower proportions of respiratory distress (15.8 vs. 71.2%, *p* < 0.001), blood transfusion (5.3 vs. 32.7%, *p* = 0.028), and oxygen administration (31.6 vs. 86.5%, *p* < 0.001) among ROP patients in Group 1 than those in Group 2 were revealed. Vaginal delivery [OR: 4.73 (1.83–12.26)] was identified as a factor associated with ROP among the infants in Group 1. Forty percent (6/15) of Type 1 ROP in this study would have been missed under the current screening criteria in China (GA ≤ 34 weeks and/or BW ≤ 2,000 g). Trends of increased incidence of Type 1 ROP and decreased BW were exhibited in the 7-year study period.

**Conclusions:**

These findings indicate that even the premature infants with late GA and large BW also have a high risk of developing ROP, especially for those delivered by vagina. The findings may provide a significant reference for ROP screening and neonatal care in South China and other regions with similar conditions.

## Introduction

Retinopathy of prematurity (ROP) is one of the leading causes of potentially preventable and treatable blindness among premature infants worldwide (1). Although the incidence of ROP is relatively low, a considerable number of ROP patients are being diagnosed in China every year due to their large population base. Furthermore, with the second-child policy announced in China in 2015 (2), the number of potential patients with ROP further increased, leading to a heavy socioeconomic burden. A neonatal screening program is one of the most effective strategies for the early detection of patients with ROP (3, 4). The screening criteria for ROP vary among countries or cities with different socioeconomic and medical conditions (5–7). Most screening criteria of ROP are set based on gestational age (GA) or birth weight (BW): GA ≤ 30 weeks and/or BW ≤ 1,500 g in the United States (US) (8), GA ≤ 32 weeks and/or BW ≤ 1,500 g in United Kingdom (UK) (9), and GA ≤ 34 weeks and/or BW ≤ 2,000 g in China (6). The incidence and risk factors of ROP within the Chinese screening criteria have been well explored (10). Recently, an increasing number of ROP patients with late GA and large BW in developing countries has been reported (11–13). Because of the limited medical resources, it is difficult to screen every baby born with late GA and large BW. Understanding the incidence and characteristics of ROP with late GA and large BW may help to adjust the screening strategy for the reduction of missed diagnoses. Liu et al. (14) used a broader screening range (GA <37 weeks and BW <2,500 g) to analyse the incidence and risk factors of ROP in Southwest China. However, they did not focus on the characteristics of ROP with late GA and large BW and analyzed the incidence of ROP according to GA and BW separately.

Furthermore, the Chinese Ministry of Health first issued guidelines on supplemental oxygen delivery policies and the prevention and treatment of ROP in 2004 (15). The guidelines for oxygenation were adjusted in 2013 after 9 years of clinical practice and exploration (16). However, few studies have focused on the changes in the incidence of ROP before and after this adjustment, which is significant for the effect evaluation, further guideline readjustment, and the prevention and treatment strategies of ROP. In this study, we focused on the incidence and characteristics of ROP patients with both late GA (≥35 and <37 weeks) and large BW (≥1,750 and <2,500 g) and presented a 7-year trend of ROP incidence in a tertiary hospital in South China. This study may provide clinical references for the improvement of ROP screening strategy and neonatal care in South China and other regions with similar socioeconomic and medical conditions.

## Materials and Methods

### Inclusion Criteria and Ethics Statement

This was a retrospective, cross-sectional study of the incidence and characteristics of ROP among premature infants over a 7-year period (from 1 January 2010 to 31 December 2016) at the Sun Yat-sen Memorial Hospital (SYSMH), one of the largest and the oldest tertiary general hospitals in Guangzhou, South China. In the database of the Medical Records Department of SYSMH, premature infants and ROP were encoded using the International Classification of Diseases, 9th Revision, Clinical Modification (ICD-9-CM) (17) and the New Rural Cooperative Medical System Version of ICD-9-CM. Premature infants were identified with the following three codes: P07.300; P07.301; and P07.302. Patients with ROP were identified with H35.100. This study followed the tenets of the Declaration of Helsinki and was approved by the institutional review board of SYSMH in Sun Yat-sen University (IRB-SYSMH-SYSU), Guangzhou, China. All infants were anonymized and de-identified before being analyzed, and this study was exempted from participant consent by the IRB-SYSMH-SYSU.

### Screening Schedule and Examination Methods

According to the “Chinese Ministry of Health guidelines on oxygenation policies and prevention and treatment of ROP” [versions of 2004 (15) and 2013 (16)] and the clinical experience of the neonatology and ophthalmology departments of SYSMH, all infants who had GA <37 weeks or BW <2,500 g were routinely examined by pediatric ophthalmologists.

The first examination was performed at 32 weeks of postmenstrual age or 4–6 weeks after birth, whichever came last. If ROP-related change was not detected at the first time point, these infants were followed up every 2 weeks until the vascularization of peripheral retina was completed. Eyes were diagnosed as Type 1 ROP if they had zone 1 ROP with plus disease; zone 1, stage 3 ROP without plus disease; or zone 2, stage 2 or 3 ROP with plus disease (18). Once Type 1 ROP was detected, infants would be referred to the Zhongshan Ophthalmic Center or the Guangzhou Women and Children's Medical Center for further treatment. Laser photocoagulation was performed within 72 h. The eyes with zone 1, stage 1 or 2 ROP without plus disease or with zone 2, stage 3 ROP without plus disease were considered as Type 2 ROP (18). If a mild ROP (Type 2 ROP or a less severe ROP that temporarily did not require treatment) was detected at the first time point, these infants were followed up every week or every other week (when the extent of ROP-related change decreased in the follow up) until the lesion regressed completely.

All fundus examinations were performed by two experienced ophthalmologists (XG and YYL) and mutually checked once ROP was detected. Pupils were dilated with 0.5% Compound Tropicamide Eye Drops (Zhuo Bi'an, Xingqi Eye Medicine Company Limited, China) before examination (usage: 3 times, one drop every 5 min). Infants were in the supine position and were held by an experienced nurse. Binocular indirect ophthalmoscopy was performed using a lid speculum, pre-set lens (+20 Dioptres) and a scleral compressor after topical anesthesia (0.5% Alcaine, Alcon, USA). Infants with ROP were classified and recorded according to the International Classification of ROP.

### Information Extraction

The number of premature infants and infants with ROP in the 7-year period were extracted. The disease information, including the stage, zone, presence or absence of plus disease, and laterality of patients with ROP, were collected. Potential risk factors of ROP were also extracted, including sex, number of fetuses, GA (between the first day of the last menstrual period and the date of birth), BW, delivery manner (vaginal or cesarean), blood transfusion, respiratory distress, and information on oxygen administration (mode of delivery and duration of oxygen administration).

### Screening Guidelines of the US, UK, China, and Current Study

To explore the appropriate screening criteria of ROP in South China, we applied the ROP screening guidelines of the US (GA ≤ 30 weeks and/or BW ≤ 1,500 g) (8), UK (GA ≤ 32 weeks and/or BW ≤ 1,500 g) (9), and China (GA ≤ 34 weeks and/or BW ≤ 2,000 g) (6) among premature infants in the current study. The numbers of premature infants who exceeded the screening criteria for ROP and the ROP patients (including Type 1 ROP) who may be missed diagnoses were recorded.

### Statistical Analysis

All of the data were entered into Microsoft Excel (Microsoft Corp., Redmond, Washington, USA) spreadsheets, sorted and analyzed by two researchers. The data were further imported into the Statistical Package for the Social Sciences (SPSS ver. 19.0, Chicago, IL, USA) for statistical analysis. All included infants were divided into two groups for the characteristic analysis of ROP with late GA and large BW: Group 1 (with both GA ≥ 35 weeks and BW ≥ 1,750 g) and Group 2 (others). The absolute frequency (*n*) and relative frequency (%) were used for quantitative variables, such as the incidence of ROP and the ratio of males to females. An independent *t*-test was used to compare GA and BW between the two groups. The Pearson chi-square test or Fisher's exact test (expected count <5) were used to compare the proportions of ROP and potential risk factors between the two groups. Binary logistic regression was used to analyze the relationships between ROP and the following factors among premature infants in Group 1, including the sex, GA, BW, multiple birth, delivery manner, blood transfusion, respiratory distress, and information of oxygen administration. The odds ratio (OR) and 95% confidential interval (CI) were used to determine the risk factors for ROP in Group 1. All tests of hypotheses were 2-tailed. The level of significance was set at *P* < 0.05.

## Results

### Incidence and Characteristics of ROP With Late GA and Large BW

A total of 911 premature infants were included, with 282 in Group 1 and 629 in Group 2. Seventy-one ROP patients were identified, with an overall incidence of 7.8%. One infant with Type 2 ROP (1/911, 0.1%) and 15 Type 1 ROP (15/911, 1.6%) infants were identified. All Type 1 ROP infants were treated by laser photocoagulation.

As shown in Table 1, both the incidences of any ROP (6.7 vs. 8.3%, *p* = 0.43) and Type 1 ROP (1.4 vs. 1.7%, *p* = 1.00) in Group 1 were comparable with those in Group 2. Group 1 infants were less likely to be diagnosed as respiratory distress (8.5 vs. 66%, *p* < 0.001) and less likely to have received blood transfusion (1.1 vs.30.2%, *p* < 0.001) or supplemental oxygen (31.6 vs. 80.6%, *p* < 0.001) than Group 2. The information on the mode of oxygen delivery among infants in two groups are shown in Supplementary Material. In Table 2, lower proportions of respiratory distress (15.8 vs. 71.2%, *p* < 0.001), blood transfusion (5.3 vs. 32.7%, *p* = 0.028), and oxygen administration (31.6 vs. 86.5%, *p* < 0.001) among ROP patients were revealed in Group 1 than those in Group 2. We compared Group 1 infants with and without any ROP (Table 3). Vaginal delivery was identified as a factor associated with ROP among infants in Group 1 (OR: 4.73, 95% CI: 1.83–12.26; *p* = 0.004 in binary logistic regression analysis).

**Table 1:** Characteristics of premature infants with both late gestational age and large birth weight.

	**Infants ≥35 weeks and ≥1,750 g**	**Infants <35 weeks or <1,750 g**	**χ^2^/*t***	***P***
Number screened	282	629	–	–
Male	50.7% (143/282)	53.7% (338/629)	0.72	0.40
Gestational age, weeks (mean, range)	35.8 (35–37.6)	32.3 (26.4–36.9)	–	–
Birth weight, grams (mean, range)	2,346 (1,750–3,200)	1,708 (640–2,950)	–	–
Any ROP	6.7% (19/282)	8.3% (52/629)	0.63	0.43
Type 1 ROP	1.4% (4/282)	1.7% (11/629)	–	1.00
Type 2 or milder ROP	5.3% (15/282)	6.5% (41/629)	0.49	0.49
Multiple birth^†^	49.3% (139/282)	46.4% (292/629)	0.64	0.42
Vaginal delivery	21.3% (60/282)	21.9% (138/629)	0.05	0.82
Blood transfusion	1.8% (5/282)	30.2% (190/629)	99.04	<0.001^#^
Respiratory distress	8.9% (25/282)	66% (415/629)	257.54	<0.001^#^
Oxygen administration	31.6% (89/282)	80.6% (507/629)	207.03	<0.001^#^
Duration of oxygen administration, days (mean, range)	6.1 (1–30)	13.8 (1–116)	−6.19	<0.001^*^

*ROP, retinopathy of prematurity*.

† *Twins or multiplets*.

# *p < 0.05, significant difference by Pearson chi-square test*.

* *p < 0.05, significant difference by independent t-test*.

**Table 2:** Characteristics of any ROP with both late gestational age and large birth weight.

	**Infants ≥35 weeks and ≥1,750 g**	**Infants <35 weeks or <1,750 g**	**χ^2^/*t***	***P***
Number of any ROP	19	52	–	–
Male	42.1% (8/19)	61.5% (32/52)	2.14	0.14
Gestational age, weeks (mean, range)	35.8 (35–36.7)	30.5 (26.4–36)	–	–
Birth weight, grams (mean, range)	2,331 (1,920–2,780)	1,492 (700–2,280)	–	–
Type 1 ROP	21.1% (4/19)	21.2% (11/52)	–	1.00
Multiple birth^†^	36.8% (7/19)	48.1% (25/52)	0.71	0.40
Vaginal delivery	52.6% (10/19)	44.2% (23/52)	0.40	0.53
Blood transfusion	5.3% (1/19)	32.7% (17/52)	–	0.028^##^
Respiratory distress	15.8% (3/19)	71.2% (37/52)	17.34	<0.001^#^
Oxygen administration	31.6% (6/19)	86.5% (45/52)	20.77	<0.001^#^
Duration of oxygen administration, days (mean, range)	2 (1–3)	14.4 (1–45)	−3.05	0.02^*^

*ROP, retinopathy of prematurity*.

† *Twins or multiplets*.

# *p < 0.05, significant difference by Pearson chi-square test*.

## *p < 0.05, significant difference by Fisher's exact test*.

* *p < 0.05, significant difference by independent t-test*.

**Table 3:** Comparisons of any ROP and those without ROP among premature infants with both late gestational age and large birth weight.

	**Any ROP**	**Premature infants without ROP**	**OR (95% CI)**	***P***
Number of ROP	19 (6.7%)	263 (93.3%)	–	–
Male	42.1% (8/19)	51.3% (135/263)	0.69 (0.27–1.77)	0.44
Gestational age, weeks (mean, range)	35.8 (35–36.7)	36.4 (32.4–38.6)	0.96 (0.44–2.10)	0.92
Birth weight, grams (mean, range)	2,331 (1,920–2,780)	2,347 (1,750–3,200)	1.00 (0.99–1.00)	0.83
Multiple birth^†^	36.8% (7/19)	50.2% (132/263)	0.63 (0.24–1.64)	0.34
Vaginal delivery	52.6% (10/19)	19.0% (50/263)	4.73 (1.83–12.26)	0.001
Respiratory distress	15.8% (3/19)	8.4% (22/263)	2.05 (0.56–7.60)	0.28
Blood transfusion	5.3% (1/19)	1.2% (4/263)	3.60 (0.38–33.88)	0.26
Oxygen administration	31.6% (6/19)	31.6% (83/263)	1.00 (0.37–2.73)	0.99
Duration of oxygen administration, days (mean, range)	2 (1–3)	4.3 (1–30)	0.77 (0.48–1.23)	0.27

*Except vaginal delivery, the P-values of all factors were much larger than 0.05 by univariate analysis, so the multivariate analysis were not further performed*.

*ROP, retinopathy of prematurity; OR, odds ratio; CI, confidential interval*.

† *Twins or multiplets*.

### Applicability of the Screening Guidelines of the US, UK, and China in the Present Study

**Figure 1:**
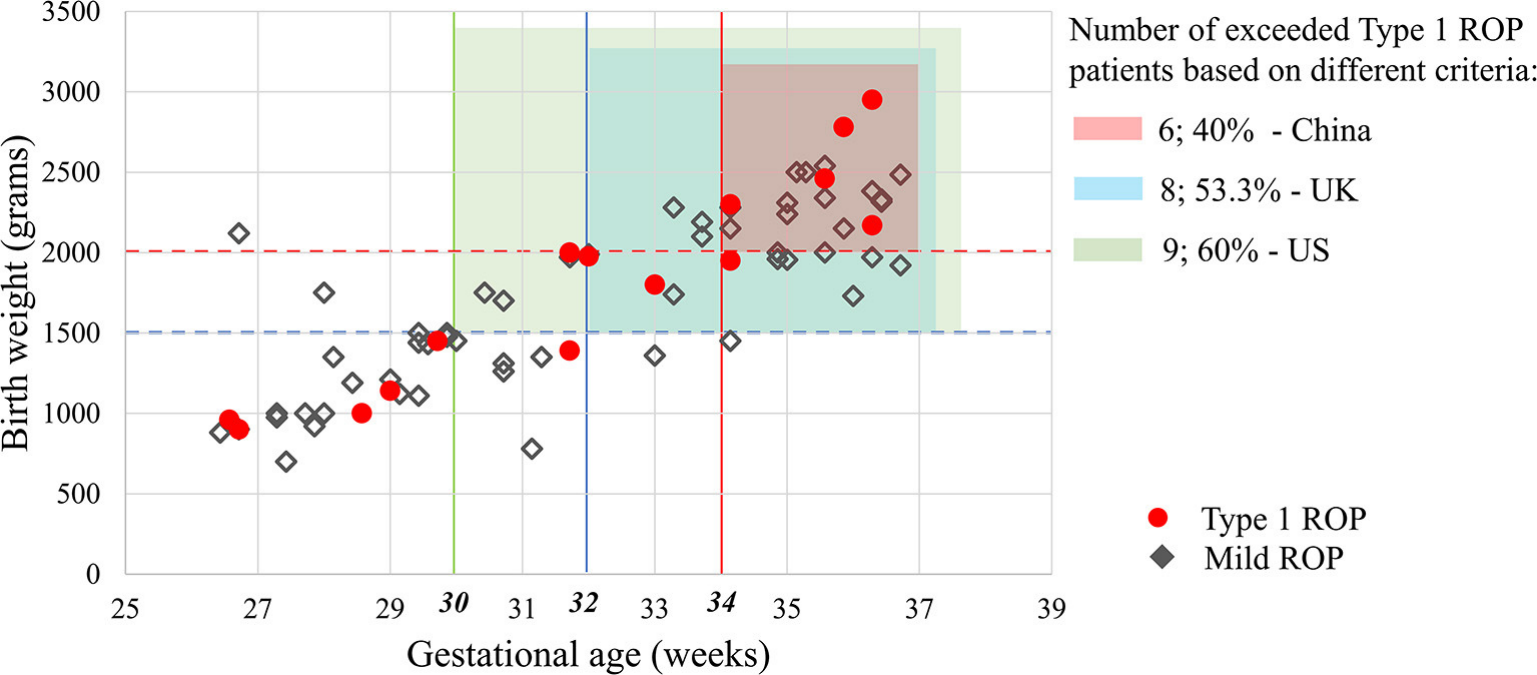
Applicability of the screening guidelines of the US, UK and China in the present study. Based on different screening criteria in these countries, 25.4–52.1% of infants with ROP and 40–60% of Type 1 ROP patients in this study would have been missed. Mild ROP was defined as Type 2 ROP or a less severe ROP that did not require treatment in this study.

The numbers of premature infants who exceeded the screening criteria and the ROP infants (any ROP and Type 1 ROP) who may be missed are shown in Figure 1 and Table 4. Six infants with Type 1 ROP in this study would have been missed under the current screening criteria in China.

**Table 4:** Numbers of infants exceeding the screening guidelines of the US, UK, and China and the ROP patients who may be missed.

	**Exceeding infants**	**Any ROP**	**Stage (1:2:3)**	**Type 1 ROP**
**US** (GA ≤ 30 weeks and/or BW ≤ 1,500 g)	703 (77.2%)	37 (52.1%)	25:11:1	9 (60%)
**UK** (GA ≤ 32 weeks and/or BW ≤ 1,500 g)	633 (69.5%)	31 (43.7%)	21:10:0	8 (53.3%)
**China** (GA ≤ 34 weeks and/or BW ≤ 2,000 g)	321 (35.2%)	18 (25.4%)	11:7:0	6 (40%)

*In these countries, infants with an unstable clinical course would also be screened even they have a BW or GA a little exceed the national screening criteria. These infants had not been accounted for in the analysis due to the uncertainty, so the estimates for percent of infants missed in this study is an upper bound*.

*ROP, retinopathy of prematurity*.

### 7-Year Incidence of Type 1 ROP

Table 5 shows that the number of screened premature infants increased from 2010 to 2016. Type 1 ROP was not detected until 2013, and the incidence increased from 1.8% in 2013 to 3.4% in 2016. A downward trend of BW in Type 1 ROP from 2013 to 2016 was revealed.

**Table 5:** 7-year trends of type 1 ROP.

**Year**	**Number screened**	**Any ROP, (*N*, %)**	**Type 1 ROP, (*N*, %)**	**GA of type 1 ROP (mean, range)**	**BW of type 1 ROP (mean, range)**
2010	69	0	0	0	0
2011	84	2 (2.4%)	0	0	0
2012	140	8 (5.7%)	0	0	0
2013	113	13 (11.5%)	2 (1.8%)	34.3 (33–35.6)	2,130 (1,800–2,460)
2014	161	9 (5.6%)	3 (1.9%)	32.5 (29.7–35.9)	2,069 (1,976–2,180)
2015	146	8 (5.5%)	3 (2.1%)	34.0 (31.7–36.3)	2,040 (1,950–2,170)
2016	203	31 (15.3%)	7 (3.4%)	30.4 (26.6–36.3)	1,520 (900–2,950)

*ROP, retinopathy of prematurity; N, number; BW, birth weight; GA, gestational age*.

## Discussion

The incidence of any ROP in China has been well investigated, ranging from 9.4% (10) to 17.8% (11). However, the incidence and characteristics of ROP among premature infants with late GA and large BW remain unclear. In this study, we found that the incidences of any ROP were 6.7% among infants with GA ≥35 weeks and BW ≥ 1,750 g and 8.3% among infants with a younger GA and smaller BW, both of which were lower than the previously reported ROP incidence in other regions of China. Liu et al. (14) revealed an overall ROP incidence of 12.8% at a children hospital located in Southwestern China from 2009 to 2012 using the same screening criteria. Since the ROP incidence varied according to geographical regions, the relatively higher degree of socioeconomic development and the quality of neonatal care of Guangzhou city may be a contributing factor to the low incidence of ROP. Furthermore, the differences of hospital type and study period with distinct patient conditions and medical development levels may also contribute to the different reported ROP incidences. In addition, a few premature infants, who died, were transferred or discharged prior to being screened, would have been missed the ROP screening, which may cause underestimation of the ROP incidence. The premature infants with late GA and large BW also developed any ROP and Type 1 ROP in equal proportions to those with a younger GA and smaller BW in the current study, even though much lower proportions of blood transfusion, respiratory distress, and oxygen administration were observed in the larger infants. Furthermore, 40% of patients with Type 1 ROP in our study would have been missed by the current screening criteria in China even in units where neonatal care is improving. Because ROP patients can progress to retinal detachment, missed diagnosis of ROP may result in irreversible visual impairments in the critical period of visual development. Some eyes with mild ROP can also develop to Type 1 ROP in <1 week (18). Our findings indicate that even the premature infants with late GA and large BW also have a similar risk of developing ROP (19) and should be screened.

A low-cost ROP screening could potentially reduce the disease burden of the family and the healthcare system costs. However, screening all the premature infants with late GA and large BW is stressful and costly, how to balance the risk of missed diagnosis and the reduction of screening number of infants is clinically significant. In the current study, besides conventional risk factors of blood transfusion and respiratory distress (20), vaginal delivery was identified as a factor associated with ROP among premature infants with late GA and large BW. Similar findings were previously reported in premature infants with very low birth weight in both Taiwan of China (21) and Italy (22). A relationship between perinatal hypoxia and ROP has been previously reported (23), and the pressure dynamics of vaginal delivery may cause hyperoxia-hypoxia imbalance of cerebral vessels of fetus (22). Furthermore, premature neonates are easily to be infected by vaginal microorganisms (such as Ureaplasma urealyticum and Candida spp.) during vaginal delivery, which is occasionally related to increased risk of retinal neovascularization (24) and ROP (25, 26). Therefore, ROP screening is recommended to those delivered by vagina among the premature infants with late GA and large BW.

In a 7-year study period before and after the policy adjustment of guidelines on supplemental oxygen delivery, the number of premature infants screened and the incidence of Type 1 ROP increased by years, with a downward trend of BW. These findings suggest that the quality of neonatal care has improved over time, and it may be attributed to the decreases of transcutaneous oxygen saturation and the volume fraction of oxygen, and the use of pulmonary surfactant (16).

There are several limitations in this study. First, this is a single center study conducted in a neonatal department of one tertiary general hospital with a relatively small sample size, especially for Type 1 ROP. Second, the proportions of vaginal delivery were very low in both two groups, which suggested that these infants may have other health issues apart from prematurity. The included premature infants may be more representative for those with sicker health conditions in the neonatal department of tertiary hospitals. Therefore, the association between vaginal delivery and ROP revealed in this study should be interpreted with caution. In order to develop suitable ROP screening criteria in South China, future multicentre studies with a larger sample size in other facilities with different health care levels and located in other areas should be used for analyzing the characteristics of ROP patients, how rates of Type 1 ROP vary and change over time, and the characteristics of the infants affected. In addition, limited by the retrospective nature of the study, information on the socioeconomic condition of patient families among premature infants was unavailable. This important information will be investigated in our following prospective studies.

The incidences of any ROP and Type 1 ROP among premature infants with late GA and large BW were comparable with those of infants within the screening criteria in China. Furthermore, the increased number of infants screened, increased proportion of Type 1 ROP, and decreased BW of Type 1 ROP during the 7-year study period probably reflect improvements in neonatal care following the new revised policies in 2013, which have led to greater survival of extremely premature infants, but more mature infants are still being exposed to risk factors for ROP. Findings from this study suggest that screening criteria in China should be expanded to include more mature infants. ROP screening is recommended to those delivered by vagina among the premature infants with late GA and large BW.

## Data Availability Statement

The original contributions presented in the study are included in the article/Supplementary Material, further inquiries can be directed to the corresponding author.

## Ethics Statement

The studies involving human participants were reviewed and approved by the Institutional Review Board of Sun Yat-sen Memorial Hospital. Written informed consent for participation was not provided by the participants' legal guardians/next of kin because: all infants were anonymized and de-identified before being analyzed and this study was exempted from participant consent by the Institutional Review Board of Sun Yat-sen Memorial Hospital.

## Author Contributions

DL, YLa, XG, and YLi contributed to the conception or design of the work. YLi, XG, LW, ZL, and YZ contributed to the acquisition of data. DL, LW, and DY contributed to the analysis. DL, XG, and YLi contributed to the interpretation of data for the work. DL and XG contributed to drafting the work and revising it critically for important intellectual content. YLa agree to be accountable for all aspects of the work. All authors gave final approval of the version to be published.

## Funding

This study was funded by the National Natural Science Foundation of China (82000946), Natural Science Foundation of Guangdong Province (2021A1515012238), and Science and Technology Planning Project of Guangzhou (DL). The funders played no role in the study design, data collection and analysis, decision to publish, or preparation of the manuscript.

## Conflict of Interest

The authors declare that the research was conducted in the absence of any commercial or financial relationships that could be construed as a potential conflict of interest.

## Publisher's Note

All claims expressed in this article are solely those of the authors and do not necessarily represent those of their affiliated organizations, or those of the publisher, the editors and the reviewers. Any product that may be evaluated in this article, or claim that may be made by its manufacturer, is not guaranteed or endorsed by the publisher.
