# Supplementary material for: Incidence and Characteristics of Retinopathy of Prematurity Patients With Late Gestational Age and Large Birth Weight in South China
Source: Front Med (Lausanne). 2022 Mar 3;9:712759. doi: 10.3389/fmed.2022.712759 (PMC8927732; doi:10.3389/fmed.2022.712759)
Supplement: Supplementary file 1 [file Data_Sheet_1.docx]

**Table S1**. Mode of oxygen administration of premature infants with both late gestational age and large birth weight.

|  | **Infants ≥35 weeks and ≥1750 grams** | **Infants <35 weeks or <1750 grams** |
| --- | --- | --- |
| Number of infants with oxygen administration | 89 | 507 |
| nasal cannula | 48 (54%) | 62 (12.2%) |
| CAPA | 35 (39.3%) | 345 (68.0%) |
| p-cmv | 0 | 42 (8.3%) |
| p-simv | 1 (1.1%) | 16 (3.2%) |
| biPAP | 0 | 14 (2.8%) |
| Unclear | 5 (5.6%) | 28 (5.5%) |

Notes: CPAP: Continuous Positive Airway Pressure; biPAP: bi-level positive airway pressure; P-simv: Pressure-Synchronized Intermittent Mandatory Ventilation; p-cmv: Pressure-Control Mechanical Ventilation.

**Table S2.** Comparisons of the mode of oxygen administration between ROP patients and those without ROP among premature infants with both late gestational age and large birth weight.

|  | **ROP patients** | **Infants without ROP** |
| --- | --- | --- |
| Number of infants with oxygen administration | 6 | 83 |
| nasal cannula | 3 (50%) | 45 (54.2%) |
| CAPA | 3 (50%) | 32 (38.6%) |
| p-simv | 0 | 1 (1.2%) |
| Unclear | 0 | 5 (6.0%) |

Notes: CPAP: Continuous Positive Airway Pressure; P-simv: Pressure-Synchronized Intermittent Mandatory Ventilation.
